# Membrane transporter genes predict chemoradiotherapy response in patients with cervical cancer

**DOI:** 10.31744/einstein_journal/2025AO1154

**Published:** 2025-07-30

**Authors:** Natália Gregório Custódio, Fábio Ribeiro Queiroz, Angelo Borges de Melo, Brenda Martins Cavalcante, Laurence Rodrigues do Amaral, Telma Maria Rossi de Figueiredo Franco, Matheus de Souza Gomes, Vasco Ariston de Carvalho Azevedo, Letícia da Conceição Braga, Paulo Guilherme de Oliveira Salles, Wander de Jesus Jeremias

**Affiliations:** 1 Universidade Federal de Minas Gerais Instituto de Ciências Biológicas Laboratory of Cellular and Molecular Genetics Belo Horizonte MG Brazil Laboratory of Cellular and Molecular Genetics, Instituto de Ciências Biológicas, Universidade Federal de Minas Gerais, Belo Horizonte, MG, Brazil.; 2 Instituto Mário Penna Research and Teaching Center Translational Research Laboratory Belo Horizonte MG Brazil Translational Research Laboratory, Research and Teaching Center, Instituto Mário Penna, Belo Horizonte, MG, Brazil.; 3 Universidade Federal de Uberlândia Bioinformatics and Molecular Analysis Laboratory Patos de Minas MG Brazil Bioinformatics and Molecular Analysis Laboratory, Universidade Federal de Uberlândia, Patos de Minas, MG, Brazil.; 4 Universidade Federal de Ouro Preto Faculdade de Farmácia Laboratory of Experimental Pharmacology Ouro Preto MG Brazil Laboratory of Experimental Pharmacology, Faculdade de Farmácia, Universidade Federal de Ouro Preto, Ouro Preto, MG, Brazil.

**Keywords:** Uterine cervical neoplasms, Drug resistance, neoplasms, Biomarkers, Chemoradiotherapy, Membrane transport proteins, Carrier proteins, Transcriptome, Adenosine triphosphatases, Solute carrier proteins

## Abstract

**Introduction:**

Cervical cancer is the fourth most common cancer in women worldwide. Resistance to chemoradiotherapy in cervical cancer has been widely associated with membrane transport-related genes, particularly those encoding efflux transport proteins, such as the ATP-binding cassette family members (including P-glycoprotein), which act by expelling chemotherapeutic agents from tumor cells, as well as solute carrier proteins, whose expression impairs the uptake of antineoplastic drugs by cancer cells.

**Objective:**

This study aimed to identify specific membrane transport-related gene expression profiles as potential biomarkers for predicting chemoradiotherapy response in cervical cancer.

**Methods:**

Cervical biopsies were collected from 31 patients (21 responders and 10 non-responders) at *Hospital Luxemburgo - Instituto Mário Penna.* Fluorescence-activated cell sorting was used to separate non-stem cancer cells from cervical cancer biopsies. cDNA libraries from the 21 responders and 10 non-responders were sequenced using the Illumina platform. Expression analysis was performed using R and the DESeq2 package, with differentially expressed genes identified based on log fold change >1 or <-1 and padj ≤0.05. WEKA software and decision tree methods were used to analyze membrane transporters.

**Results:**

The results revealed two major gene groups with contrasting differentially expressed genes profiles. The first group, comprising *SLC35* and *ATP13*, was overexpressed in non-responders, while the second group, consisting of *SLC25* and *ATP6*, was overexpressed in responders. Decision tree analysis revealed that *ATP1B3* and *SLCOB3* expression profiles accurately classified patients into responder and non-responder groups with 90% accuracy, indicating that *ATP1B3* and *SLCOB3* are potential predictors of chemoradiotherapy response.

**Conclusion:**

Our results strongly suggest the presence of a candidate gene signature comprising *ATP1B3* and *SLCO1B3* that holds predictive value for chemoradiotherapy response in cervical cancer.

## In Brief

This study aimed to explore membrane transporter gene expression as a predictive biomarker of chemoradiotherapy response in cervical cancer. The differential expression of *ATP1B3* and *SLCO1B3* accurately classified patients as responders or non-responders with 90% accuracy, highlighting their potential for personalized treatment strategies.

## Highlights

■ Two gene groups with contrasting expression profiles were identified.
■ The *ATP1B3* and *SLCOB3* gene profiles classified patients with 90% accuracy.
■ The *ATP1B3* and *SLCOB3* gene signature is a potential predictor of treatment response.

## Graphical abstract

**Figure:**
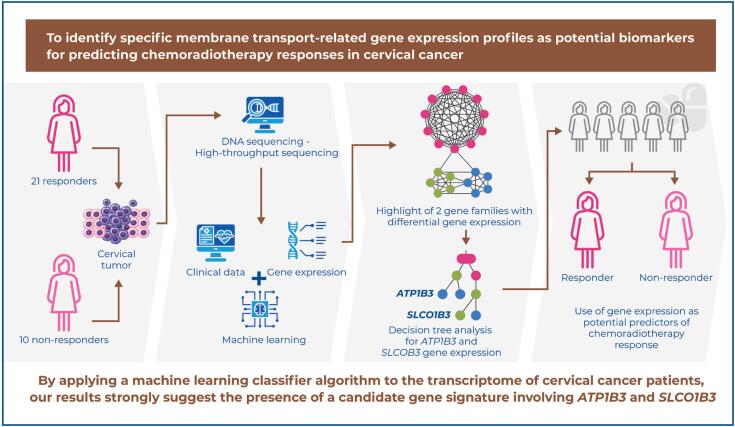

## INTRODUCTION

Cervical cancer (CC) is the fourth most common cancer among women, accounting for approximately 6.5% of all cancer cases and causing nearly 8% of cancer-related deaths worldwide.^(1)^ Squamous cell carcinoma and adenocarcinoma are the primary types of CC, accounting for approximately 70% and 25% of cases, respectively, with varying prevalence across regions.^(2)^ Low- and middle-income countries experience significantly higher incidence and mortality rates, with over 85% of new cases and 80% of CC-related deaths occurring in these countries due to less effective screening programs and treatment infrastructure.^(3)^

Exposure to human papillomavirus (HPV) is a major risk factor for CC .^(4)^ Additionally, reproductive, sexual, and behavioral factors, such as high parity, multiple sexual partners, smoking, and low socioeconomic status, may also contribute to CC risk.^(5)^

The survival rate of CC depends on the stage at diagnosis. Early detection and treatment have resulted in a 5-year survival rate of 92%. However, at more advanced stages, where CC has metastasized to adjacent tissues, organs, and regional lymph nodes, the 5-year survival rate is 58%. Further, in patients with distant metastasis, the 5-year survival rate is only 17%.^(6)^

Despite progress in CC screening technology and HPV vaccine delivery, the overall survival rate and treatment response remain unsatisfactory.^(7)^ Additionally, although standard treatment options, such as chemotherapy, surgery, radiation therapy, and brachytherapy, are beneficial,^(8)^ some tumors may develop resistance to treatment, making therapeutic decisions challenging. Moreover, the molecular mechanisms underlying chemoradiation resistance in CC are not fully understood.^(9)^

Membrane transport proteins show promise as potential prognostic biomarkers for cancer as they play critical roles in determining cell fate, survival, death, and malignant transformation.^(10)^ Moreover, they are involved in the regulation of the absorption and distribution of chemotherapeutics in tissues, which can affect tumorigenesis and chemoradiation resistance.^(11)^

The solute carrier (*SLC*) gene superfamily, the second-largest family of membrane transporter proteins, is essential for maintaining homeostasis by absorbing solutes that do not freely diffuse across biological membranes and transporting drugs.^(12)^ *Solute carrier* genes are expressed in various cancer types and may exhibit differential expression in malignant and non-malignant tissues.^(13)^ Different expression patterns of these transporters have been associated with metastatic cancer, suggesting their potential as therapeutic targets and prognostic indicators for drug-resistant metastatic disease.^(14)^

Adenosine triphosphatases (ATPases) and adenosine triphosphate synthases (ATP synthases) are involved in energy metabolism, and changes in their functions are associated with tumor angiogenesis, metastasis, and drug resistance.^(15)^ ATP-binding cassette (*ABC*) transporters, solute transporters, and ATPase membrane protein superfamilies have been identified as direct influencers of platinum-based drug pharmacology^(16)^ and may affect drug accumulation in resistant cells by increasing efflux or decreasing drug absorption.^(17)^

One of the primary challenges in cancer treatment is tailoring the therapy for individual patients. However, there are limited tools for accurately predicting patient's response or resistance to specific treatments. To address this, machine learning (ML) tools for facilitating biomarker development and supporting medical decision-making have been developed and gained prominence in clinical practice.^(18)^ Using these ML algorithms, sequencing data can be harnessed to predict treatment responses based on gene expression.

## OBJECTIVE

To identify membrane transporter genes and correlate their differential expression patterns with chemoradiation therapy resistance in patients with cervical cancer, with the aim of enhancing decision making for a more efficient and targeted therapy.

## METHODS

### Patient recruitment and sample selection

Patients with CC (squamous cell carcinoma or adenocarcinoma) with no previous history of immunological diseases or CC diagnosis were recruited at the *Hospital Luxemburgo - Instituto Mário Penna* for the study. Thirty-one patients who met the above criteria were enrolled. Tumor staging followed the recommendations of the International Federation of Gynaecology and Obstetrics (FIGO - *Federação Internacional de Ginecologia e Obstetrícia*). Cervical biopsies were collected from these patients before initiating chemoradiotherapy. Health monitoring of the patients was performed for the latest eight months through imaging and cytohistological examinations. Patients with cervical lesions during this period were classified as non-responders (NR) to treatment (10 patients), while those with no cervical lesions were classified as responders (R) (21 patients).

This study complied with the guidelines of the Institutional Research Ethics Committee of the *Instituto Mário Penna* (CAAE: 82703418.8.0000.5121; #4.914.054), and informed consent was obtained from all patients. Further details regarding the sample selection process can be found in our previous study.^(19)^

### Non-stem cervical cancer cell sorting by flow cytometry

Previously,^(19)^ we provided a comprehensive explanation of the non-stem cell sorting process. Samples were fragmented and preserved in a specialized cryoprotective solution. Subsequently, flow cytometry was used to separate stem and non-stem cells based on specific criteria, including fluorescent signals emitted and the appearance and size of granules.

### cDNA synthesis and sequencing library preparation

RNA was extracted from non-stem cells and converted into cDNA using the SMART-Seq v4 Ultra Low Input RNA Sequencing Kit (Takara Bio, CA, USA). Successful reverse transcription was confirmed and cDNAs with the highest quality parameters were chosen for library preparation. Sequencing was conducted on the NextSeq® 550 sequencer (Illumina, CA) using a NextSeq® 500/550 High Output Kit v2. For further information on these procedures, please refer to Zuccherato et al.^(19)^ The sequences used are available in the SRA database under accession number PRJNA812529.

### Transcriptome analysis

The RNA-Seq files were subjected to an initial quality control assessment using the FastQC tool (https://www.bioinformatics.babraham.ac.uk/projects/fastqc/). The obtained reads were then subjected to adapter trimming and filtering of low-quality sequences (Phred score ≤35) using Cutadapt.^(20)^ Subsequently, a second quality control check was conducted on the trimmed sequences using FastQC. The read sequences were aligned to the human reference genome (Homo sapiens. GRCh38.83) using the STAR software.^(21)^

### Analysis of differentially expressed genes

Differences in non-stem cervical cancer cell (NSCC) mRNA expression between the R and NR patient groups were analyzed using the "Bioconductor DESeq2" package in the R program.^(22)^ Raw sequences were filtered and normalized with HTSFilter^(23)^ using the default normalization parameters of DESeq2. Genes with an adjusted p≤0.05 and logFoldChange >1 or <-1 were considered differentially expressed.

### Gene ontology, differentially expressed genes selection, and heatmap visualization

Gene ontology analysis was performed using the EnrichPlot^(24)^ and ClusterProfiller R^(25,26)^ packages. Initially, all the differentially expressed genes (DEGs) obtained from RNA-Seq were analyzed and then the most representative ontologies were selected. Specifically, DEGs associated with the transport process were identified, and their relationships with biological process (BP) and molecular function (MF) terms were explored. Only GO terms with a padj value ≤0.05 were considered relevant.

The data selected based on the GO analysis were then used to generate heatmaps using the "pheatmap" R package (version 1.0.12). For heatmap generation, the search terms "*SLC*," "*ATP*," "*ABC*," and "*OTC*" were used to focus on relevant information.

### Machine learning algorithms, progression-free survival, and survival signature

Normalized counts of DEGs related to membrane transporters were used to construct decision trees using WEKA software (Waikato Environment for Knowledge Analysis, version 3.6.11; University of Waikato, New Zealand).^(27)^ The classifier algorithms were trained to distinguish patients as R or NR to treatment. Clinical characteristics, including treatment outcomes, metastasis, and FIGO classification, were considered.

To evaluate the classification accuracy and assess the model's generalizability, leave-one-out cross-validation (LOOCV) was performed. Additionally, hazard ratio (HR) analysis and Kaplan−Meier plots with log rank (Mantel−Cox) were conducted to explore the potential association between the expression profiles of *SLCO1B3* and *ATP1B3* and progression-free survival (PFS) using SPSS software (version 20, IBM, USA).

## RESULTS

### Clinicopathological characteristics of the cohort

Table 1 provides a detailed description of the clinicopathological characteristics of the study patients, including histological tumor type, staging, type, and duration of treatment.

**Table 1:** Clinicopathological characteristics of the patients with cervical cancer

Characteristics		n (%)
Diagnosis		
	Adenocarcinoma	1 (3.23)
	Squamous cell carcinoma	30 (96.77)
Histological grade		
	II	19 (61.29)
	III	12 (38.71)
FIGO classification		
	IIA	1 (3.23)
	IIB	13 (41.93)
	IIIB	17 (54.84)
Parametrial involvement		
	Unilateral	9 (29.03)
	Bilateral	21 (67.74)
	Free	1 (3.23)
Vaginal involvement		
	Present	27 (87.10)
	Absent	2 (6.45)
	Not applicable	2 (6.45)
Anticancer agent		
	Cisplatin	30 (96.77)
	Carboplatin	1 (3.23)
Status after 8 months of treatment		
	Responder	20 (64.52)
	Non-responder	11 (35.48)
Metastasis		
	After 4 months of treatment	
	Present	2 (6.45)
	Absent	29 (93.55)
After 8 months of treatment		
	Present	9 (29.03)
	Absent	18 (58.07)
	Not applicable	4 (12.90)

### Differentially expressed genes and gene ontology analyses

**Figure 1:**
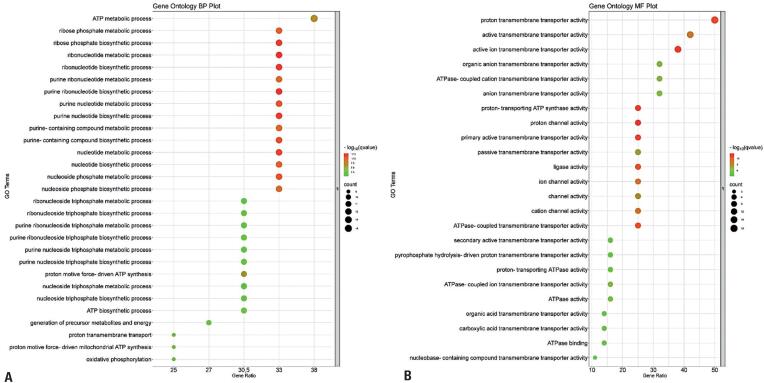
Graphical representation of the main GOs classified according to gene count. The parameters used were the ‘-log10 (qvalue)’, ‘Gene count’, number of enriched genes in a GO term, and the ‘Gene ratio’, which is the percentage of the selected DEGs in the given GO term. A) Terms related to biological processes. B) Terms related to molecular functions

GO: gene ontology; BP: biological process; MF: molecular function.

Comparison of NSCC samples between R and NR patients revealed a total of 2,519 DEGs based on the criteria: log2FoldChange >1 or <-1 and padj value ≤0.05. Among these DEGs, 41 were annotated as membrane transporters in terms of MF. Gene ontology enrichment analysis of the DEGs revealed 390 terms related to BPs, with the group related to transport in the cell membrane being the most prominent. Further, analysis of the selected DEGs revealed that a significant portion of the terms in the BP category were associated with cellular energy metabolism. Notably, essential functions, such as oxidative phosphorylation, ATP synthesis, and nucleotide metabolic processes, were highlighted (Figure 1A).

In terms of MF, 50 relevant terms were identified. As expected, all the terms were related to the transport of various substrates, with proton transmembrane transporter activity, ATPase-coupled transmembrane transporter activity, and active transmembrane transporter activity being the primary activities (Figure 1B). Consequently, these genes were selected as the focal point for the study.

Among the filtered DEGs, four primary membrane transporters were identified: *SLC, ABC*, *P-type ATPases*, and *ATP synthases*. Thirteen *SLC* genes, namely *SLC25A5, SLC25A39, SLC25A1, SLC9A3R1, SLC1A2, SLCO1B3, SLC2A4RG, SLC38A2, SLC49A4, SLC35G3, SLC25A6, SLC35G5*, and *SLC5A4P1*, showed differential expression, as did 27 genes belonging to the ATP metabolism familes*: ATPases* and *ATP synthases*. The only gene upregulated in NR patients was *ABCA10*, which belongs to the *ABC* family.

**Figure 2:**
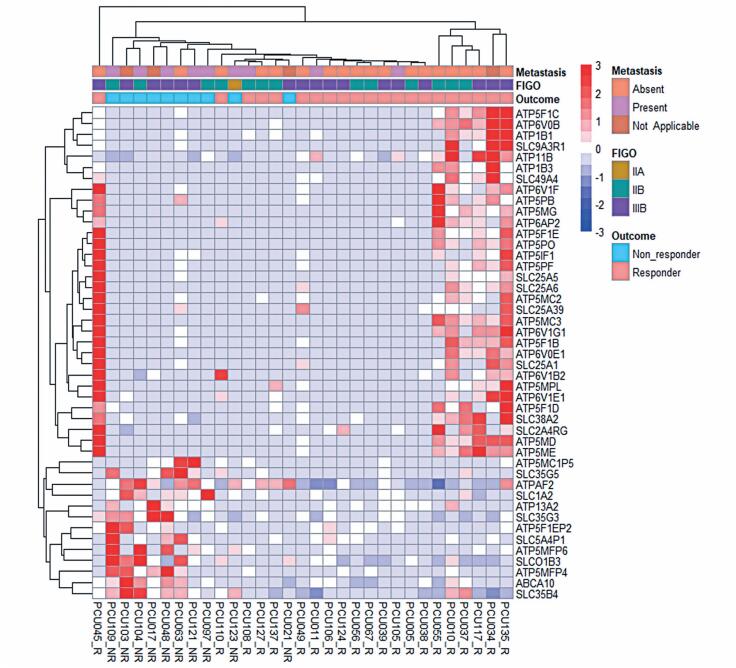
Heatmap showing the membrane transport-related DEGs (adjusted p≤0.05) in patients with cervical cancer. Responders (R; n=21) are represented in salmon pink and non-responders (NR; n=10) in blue. Other features, such as presence of metastasis and FIGO classification, are also detailed in color in the map legend

The heatmap representing the expression levels of the transporters (Figure 2) was constructed using the presence or absence of metastasis, FIGO staging, and patient outcome (R or NR) as factors for gene clustering. Heterogeneous profiles were observed across genes. The patients were segregated into two large groups with contrasting differential expression profiles, consistent with the response to chemoradiation treatment. These groups exhibited opposing gene expression patterns in R and NR patients, with the same genes being under-expressed in R and overexpressed in NR and vice versa.

### Machine learning algorithms, progression-free survival, and survival signature

**Figure 3:**
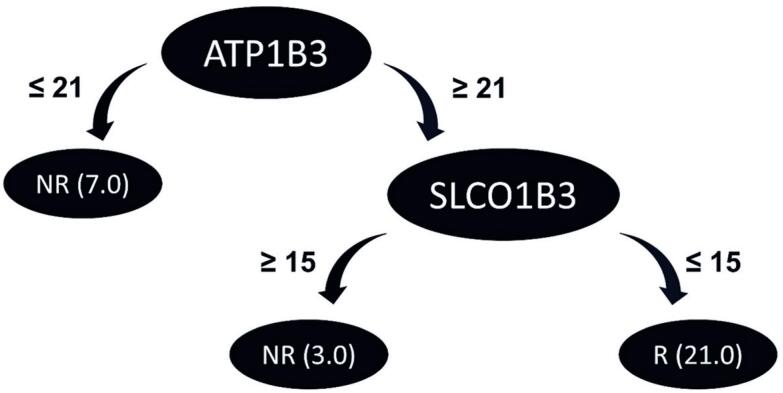
Analysis of the decision tree via machine learning, demonstrating that only the expression of the *ATP1B3* and *SLCO1B3* genes is necessary for segregating the patients into responders or non-responders. The *ATP1B3* gene alone was able to segregate 7 of the 10 non-responders with counts equal to or less than 21, while the *SLCO1B3* gene segregated the rest of the patients, using counts equal to or ≤15 as a criterion

The decision tree (Figure 3) demonstrated the efficacy of the *ATP1B3* and *SLCO1B3* genes in classifying patients as R and NR to treatment, with gene expression levels serving as a crucial determinant of patient segregation. The results revealed that the algorithm, relying solely on the *ATP1B3* gene, accurately identified seven of ten NR (70%). For the remaining patients, the differential expression of the *SLCO1B3* gene successfully classified them into R and NR. Overall, the algorithm achieved 31/31 correct classification (100%) using the complete dataset (full training). During evaluation using the LOOCV method, the algorithm correctly classified 28/31 cases, resulting in an accuracy of 90.32%.

R: responders; NR: non-responders.

**Figure 4:**
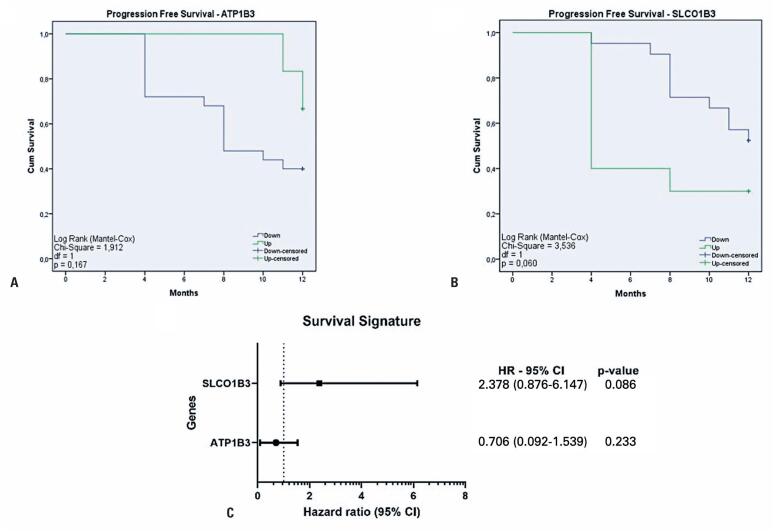
A) Kaplan−Meier plot of progression-free survival for patients with cervical cancer identified by gene expression profiling of *ATP1B3*. B) Kaplan−Meier plot of progression free survival for patients with cervical cancer identified by gene expression profiling of *SLCO1B3.* C) Estimation of the hazard ratio (HR) for *SLCO1B3* and *ATP1B3* genes signatures in relation to patient survival. A vertical line at HR=1 indicates no difference in risk. HR values above 1 indicate higher risk, while values below 1 indicate lower risk

The relationship between gene expression profiles and PFS was illustrated using Kaplan−Meier plots, and HR analysis was conducted for survival gene signatures. However, no statistically significant differences were observed in both analyses. As shown in figure 4A, patients with upregulated *ATP1B3* expression displayed a higher PFS (p=0.17). Conversely, figure 4B shows that patients with downregulated *SLCO1B3* expression had the highest PFS (p=0.06). Additionally, Figure 4C indicates that high expression of the *SLCO1B3* gene signature was associated with an increased risk of mortality, with an HR of 2.38 (95%CI= 0.876−6.147, p=0.086). Conversely, high expression of the *ATP1B3* gene was associated with a decreased risk of mortality, with an HR of 0.706 (95%CI= 0.092−1.539, p=0.233).

## DISCUSSION

The lack of biomarkers to guide medical teams in tailoring treatments for individual patients based on the intrinsic characteristics of tumors poses a significant challenge. Chemoradiation resistance remains a major obstacle in the treatment of CC. Tumor cells develop resistance to antineoplastic agents through molecular mechanisms, such as changes in drug accumulation within cells, increased drug efflux activity, and promotion of DNA repair capacity, which have been studied to improve the prognosis of patients with cancer.^(28)^

This study aimed to determine the potential of membrane transport-related DEGs as biomarkers capable of supporting clinical decision-making through accurate prediction of treatment response. To achieve this, we analyzed DEG data obtained by next-generation sequencing combined with clinical data using statistical and ML methods.

The analysis of DEGs between NR and R revealed 2,519 genes, and GO analysis results demonstrated a significant enrichment of terms related to biological processes and molecular functions, particularly membrane transport activity, in 41 of these genes. Many of the investigated genes play essential roles in cellular energy metabolism, including ATP synthesis via proton motive force, nucleotide biosynthesis, oxidative phosphorylation, and cellular respiration. The most enriched families were *SLC, ATP synthases*, *ATPases*, and *ABC*.

*SLC* genes, which encode a family of over 400 membrane-bound transporters, have been widely targeted for drug development.^(29)^ *ATPases* are enzymes that hydrolyze ATP into ADP, resulting in the release of phosphate ions,^(30)^ while *ATP synthases* are mitochondrial enzymes located in the inner membrane responsible for synthesizing ATP through electron transfer gradients.^(31)^ *ABC* transporters bind to ATP and play a role in various cellular processes, including cancer resistance, antigen processing, cell division, and immunity.^(32)^

The current results revealed two groups of genes with antagonistic expression patterns between R and NR patients. The *ATP1, ATP5*, and *SLC25* genes were the most prominent among the genes associated with patient response. *ATP1* belongs to the subfamily of Na+/K+ ATPases (*NKA*), and its primary function is to transport sodium and potassium ions across the cell membrane. Alterations in the expression of isoforms of these enzymes have been implicated in the development of human cancers.^(33)^

Downregulation of the expression of *ATP1B1*, which encodes the β1 subunit of NKA, has been identified as a crucial factor in increasing the invasiveness of epithelial cancer cells and is being studied as a potential tumor suppressor.^(34)^ On the other hand, the *ATP1B3* gene, which encodes the β3 subunit of the enzyme, regulates cell adhesion and is implicated in promoting tumorigenicity through the activation of pro-inflammatory factors in gastric cancer.^(35)^

Our findings align with those of previous studies suggesting that *ATP1B1* may function as a potential suppressor of cancer progression, as evidenced by its upregulation in treatment-responsive patients without metastasis. However, the exact role of *ATP1B3* in tumor cell resistance to antineoplastic drugs requires further investigation. In colon cancer, overexpression of functional *ATP1B3* has been shown to decrease TPp53 transcriptional activity, resulting in apoptotic resistance in neoplastic cells.^(36)^ Additionally, the expression of *ATP1B3* varies among different types of cancer, with upregulation observed in hepatocellular adenomas and focal nodular hyperplasia and downregulation in liver metastasis of pancreatic cancer.^(37)^ Although there is evidence for a positive association between *ATP1B3* and tumorigenesis, our study suggests that this gene is critical for identifying patients who respond well to treatment.

The *SLC25* family supports oxidative phosphorylation and enables transport across the inner mitochondrial membrane, linking cytosolic and mitochondrial metabolism. Additionally, some members of this family may act as potential cancer biomarkers and contribute to adaptive mechanisms that promote drug resistance in tumor cells.^(38)^

*SLC25A6* induces apoptosis in human HeLa and HepG2 cells,^(39)^ while *SLC25A5* exhibits paradoxical effects on drug resistance in different types of cancer. *SLC25A5* enhances resistance to tyrosine kinase inhibitors in lung cancer and reverses resistance to immunotherapy in hepatocellular carcinoma. Furthermore, upregulation of *SLC25A5* has been associated with better overall survival in patients with colon cancer.^(40)^ Our findings support the evidence that upregulated *SLC25* family members play an important role in cancer management, as observed exclusively in R patients.

Tumor cells have an altered energy metabolism, with drug-resistant liver cells exhibiting increased mitochondrial energy metabolism and glycolysis. *ATP5*, a mitochondrial ATP synthase, plays a crucial role in catalyzing ATP synthesis via an electrochemical proton gradient during oxidative phosphorylation. Alterations in ATP5 expression affect ATP production in treatment-resistant cells.^(41)^ This study identified that overexpression of *ATP5* family members was associated with a positive treatment response and favorable prognosis in patients undergoing chemoradiotherapy.

Members of the *ATP6* family, which encodes V-ATPase components, play a crucial role in facilitating intracellular acidification and proton extrusion.^(42)^ The resulting pH gradient alteration between the intracellular and extracellular compartments can affect drug resistance mechanisms, including the lysosomal sequestration of chemotherapy drugs.^(43)^ Similar to previous analyses, this study revealed that, overexpression of *ATP6* family members could serve as a predictive biomarker for treatment response.

The other group of genes that were upregulated in NR patients was primarily represented by the *SLC35, SLCO*, and *ABC* families. While *ABC* and *SLCO* transporters are generally involved in drug absorption, distribution, metabolism, and excretion, *SLC35* genes have been linked to sugar absorption and influence vital processes, such as cell survival and homeostasis.^(44, 45)^

High expression of *ABCA* genes has been associated with drug resistance and poor patient outcomes in various cancers, including neuroblastoma, acute lymphoblastic leukemia, and prostate, lung, pancreas, and kidney cancers. Additionally, there is evidence of a connection between the *ABCA10* gene and tumor immune infiltration, making it a potential prognostic marker.^(46,47)^ Our study indicates a possible negative relationship between the expression of *ABCA10* and response to chemotherapy treatment, highlighting its potential as a predictive tool.

*SLCO* family members have been identified as targets of immunotherapies due to their impact on the pharmacokinetics of several drugs through pharmacogenomic polymorphisms.^(48)^ Herein, *SLCO1B3* expression appeared to be a potential predictive factor, as it was overexpressed only in NR patients, suggesting a probable positive relationship with cisplatin resistance. However, further studies are required to elucidate its role in CC.

The *SLC35* gene family, including *SLC35G5*, has been linked to chemoresistance in various cancers. For instance, *SLC35G5* is reportedly upregulated in cisplatin-resistant lung cancer cells compared to that in cisplatin-sensitive cells.^(49)^ Moreover, upregulation of *SLC35G3* in colorectal cancer tissues has been associated with poor patient prognosis, while *SLC35B4* gene upregulation has been observed in multi-resistant hepatocellular carcinoma cells compared to that in non-resistant cells.^(50)^ These findings further support the notion that positive regulation of the *SLC35* family genes may indicate greater chemoresistance.

To confirm the potential of transporters as predictive biomarkers for patients with CC, decision trees were constructed and analyzed by the selected DEGs using ML methods. Additionally, HRs and Kaplan−Meier analyses were performed to demonstrate the predictive value of the identified genes. While no statistical differences were observed during patient follow-up,a noticeable relationship between the expression of *ATP1B3* and *SLCO1B3* and PFS was observed, showing promising results in terms of predictive/prognostic value. Although the p-values from the Kaplan−Meier and HR analyses did not reach the significance level of 0.05, their proximity indicated their potential significance.

The primary limitations of this study were its relatively small sample size and limited follow-up time, which may have contributed to the lack of significance. The strength of this study lies in the highlight that the *ATP1B3* and *SLCO1B3* gene signatures may influence clinical decisions, such as more frequent monitoring, personalized treatment selection, or strategic interventions. Further research with larger sample sizes and extended follow-up periods is required to validate these findings.

## CONCLUSION

The results obtained from the analysis of genes encoding membrane transporters offer promising insights into their potential as predictive biomarkers for cervical cancer. However, the expression patterns of these genes in cervical cancer have not been studied. A study focusing on membrane transporters could shed light on their roles in various biochemical functions and other mechanisms, such as tumor cell proliferation, apoptosis, and cellular drug influx. This, in turn, may establish membrane transporters as valuable predictive tools for cervical cancer or as potential molecular targets for the development of anticancer drugs.

Despite the need for further investigation, our findings were supported by the strong performance of the machine learning classifier algorithm. Our study suggests the presence of a candidate gene signature comprising *ATP1B3* and *SLCO1B3* that holds predictive value for cervical cancer. These results offer a promising direction for future research and contribute to the advancement of personalized medicine for cervical cancer treatment.
